# High burden of self-reported sexually transmitted infections among key populations in Mozambique: the urgent need for an integrated surveillance system

**DOI:** 10.1186/s12879-020-05276-0

**Published:** 2020-08-27

**Authors:** Makini A. S. Boothe, Charlotte Comé, Cynthia Semá Baltazar, Noela Chicuecue, Jessica Seleme, Denise Chitsondzo Langa, Isabel Sathane, Henry F. Raymond, Erika Fazito, Marleen Temmerman, Stanley Luchters

**Affiliations:** 1 Faculty of Medicine and Health Sciences, Ghent University, Ghent, Belgium; 2 National Institute of Health, Maputo, Mozambique; 3 National STI-HIV/AIDS Control Program, Ministry of Health, Maputo, Mozambique; 4 School of Public Health, Rutgers University, Piscataway, NJ USA; 5 ICAP, Columbia University, Pretoria, South Africa; 6 Department of Obstetrics and Gynaecology, Aga Khan University, Nairobi, Kenya; 7 Department of Population Health, Aga Khan University, Nairobi, Kenya; 8 Department of Epidemiology and Preventive Medicine, Monash University, Melbourne, Australia; 9 Burnet Institute, Melbourne, Australia

## Abstract

**Background:**

Key populations - men who have sex with men (MSM), female sex workers (FSW) and people who inject drugs (PWID) – are at high risk for sexually transmitted infections (STI) given their sexual risk behaviours along with social, legal and structural barriers to prevention, care and treatment services. The purpose of this secondary analysis is to assess the prevalence of self-reported STIs and to describe associated risk factors among participations of the first Biological Behavioural Surveillance (BBS) in Mozambique.

**Methods:**

Responses from the first BBS surveys conducted in 2011–2014 were aggregated across survey-cities to produce pooled estimates for each population. Aggregate weighted estimates were computed to analyse self-reported STI prevalence. Unweighted pooled estimates were used in multivariable logistic regression to identify risk factors associated with self-reported STI.

**Results:**

The prevalence of self-reported STI was 11.9% (95% CI, 7.8–16.0), 33.6% (95% CI, 29.0–41.3), and 22.0% (95% CI, 17.0–27.0) among MSM, FSW and PWID, respectively. MSM who were circumcised, had HIV, reported drug use, reported receptive anal sex, and non-condom use with their last male partner had greater odds of STI self-report. STI-self report among FSW was associated with living in Beira, being married, employment aside from sex work, physical violence, sexual violence, drug use, access to comprehensive HIV prevention services, non-condom use with last client, and sexual relationship with a non-client romantic partner. Among PWID, risk factors for self-reported STI included living in Nampula/Nacala, access to HIV prevention services, and sex work.

**Conclusion:**

The high-burden of STIs among survey participants requires integrated HIV and STI prevention, treatment, and harm reduction services that address overlapping risk behaviours, especially injection drug use and sex work. A robust public health response requires the creation of a national STI surveillance system for better screening and diagnostic procedures within these vulnerable populations.

## Background

The World Health Organization (WHO) estimates that there are more than one million new cases of curable sexually transmitted infections (STIs) every day globally [1]. These infections – caused by Chlamydia trachomatis, *Neisseria gonorrhoeae*, Treponema pallidum, and Trichomonas vaginalis – can have a serious impact on health status including cervical cancer, pelvic inflammatory disease, infertility and adverse mental health [2, 3]. STIs can also be transmitted through mother-to-child transmission with adverse health outcomes such as stillbirth, neonatal death, low-birth-weight and prematurity, sepsis, pneumonia, neonatal conjunctivitis, and congenital deformities [2]. STIs can also dramatically increase the risk of acquiring Human immunodeficiency virus (HIV) [1]. In the most recent AIDS Indicator Survey conducted in Mozambique, self-reported STIs among the general adult population, aged 15–49, was estimated at 7% among women and 5% on men [4].

Key populations (KP), defined as men who have sex with men (MSM), female sex workers (FSW) and people who inject drugs (PWID), are vulnerable to both HIV and other STIs given their high-risk sexual and drug use behaviours, further heightened by structural barriers such as low access to quality health services, stigma and discrimination [3]. Given, their risk profile, WHO advocates for STI interventions to be targeted to these groups [5]. The WHO focuses on four STIs that are curable: syphilis, gonorrhoea, chlamydia and trichomoniasis [1]. However, in Mozambique, similar to other low- and middle-income countries, diagnostic tests are largely unavailable and STI case management is based on symptomology [2, 6].

Mozambique’s National HIV Strategic Plans (2010–2014, 2015–2019) identify STI screening as an important intervention for people living with HIV [7, 8]. Given the lack of data about KP, the Strategic Plans also called for special surveys to be conducted among MSM, FSW, and PWID to estimate HIV prevalence, assess risk factors for HIV infection, and estimate population size of these KP. The first round of bio-behavioural surveillance surveys (BBS) were conducted between 2011 and 2014 in three urban areas in Mozambique and the methodology and main results have been previously published [9–14]. Prior to the implementation of the BBS surveys, there was no data about STIs in Mozambique among KP. However, given the associated morbidity and mortality, coupled with the social, legal and structural barriers to uptake of health care services among these population groups, it is imperative that STIs are monitored and treated in order to promote general health and well-being within these populations. In addition, efforts to control STIs among KP can also reduce transmission among members of the general population who are sexual partners of KP [2].

In this context, the purpose of this secondary analysis is to assess the prevalence of self-reported STIs among participants of the first BBS surveys in Mozambique and to describe risk factors associated with STIs among these three KP groups. This analysis provides an opportunity to assess access to comprehensive HIV/STI services and to monitor the implementation of efforts aimed toward reducing STIs among KP in Mozambique. Finally, the information will provide a baseline and evidence for improving STI prevention, diagnosis and treatment services in Mozambique.

## Methods

### Survey design

The first round of BBS surveys among KPs in Mozambique were conducted in three urban centres – Maputo (MSM, FSW, PWID), Beira (MSM, FSW), Nampula (FSW) and Nampula/Nacala (MSM, PWID) – using respondent-driven sampling (RDS) [15, 16]. RDS is a probability-based peer-to-peer sampling strategy used among hard-to-reach populations. Based on social network size, weights can be computed to produce adjusted estimates representative of the target population in the geographical location where the survey is conducted. The study design for each of the surveys have been previously published and include a description of efforts to reduce bias during data collection, analysis and interpretation [9–14].

### Study population

Participants in the MSM survey were eligible for the survey if they were biologically male, at least 18 years of age, and had engaged in oral or anal sex with one or more men in the 12 months preceding the survey. Being biologically female, at least 15 years of age, and having received money in exchange for sex from someone other than a steady partner in the six months preceding the survey were required of FSW participants. Finally, eligibility criteria for PWID was not restricted by sex, but required an individual to be at least 18 years of age. All individuals who participated in the PWID survey prior to December 2013 must have injected drugs without a prescription in the 12 months preceding the survey, however, due to slow recruitment patterns, this criterion was later modified (from January 2014 onwards) to include any person who had ever injected drugs without a prescription.

All eligible participants in the three surveys needed to have lived, worked or socialized in one of the recruitment areas in the six months preceding the survey, received a valid referral coupon from a peer, and had not previously participated in the study. Participants provided separate written informed consent for both the behavioural questionnaire and biological testing; however, only consent to the behavioural questionnaire was necessary in order to be enrolled in the study. Recruitment lasted from July to November 2011 (MSM), September 2011 to March 2012 (FSW) and October 2013 to March 2014 (PWID).

### Study measures

The questionnaires for the three surveys have been published [12–14]. For the purpose of this analysis, HIV is considered separate from STIs because of the emphasis on treatable infections, consistent with WHO guidelines [1, 5]. Given the lack of laboratory confirmatory testing, questions about self-reported STI symptoms are considered a proxy for possible STI, in line with guidance for biobehavioural surveys among KP [17, 18]. Self-reported STI was defined as responding “yes” to one or more of the following questions: “*During the last six months, have you had an abnormal discharge from your vagina, anus or penis?”, “During the last six months, have you had a sore or ulcer near your vagina, anus or penis?,”* and *“In the last six months, did someone inform you that you had or could have a sexually transmitted infection?”*

### Statistical analysis

RDS-adjusted self-reported STI prevalence was computed for each population by survey city. Due to low sample size, estimates were then pooled to produce an aggregate estimate of the variable of interest for the KP group using the aggregate estimate function of RDS Analyst software [19]. RDS-weights were calculated using the RDS II estimator, which uses the individual network size to create sampling weights [20]. Unweighted pooled estimates were used to conduct bivariable and multivariable logistic regression to identify the correlates associated with the primary outcome of interest: STI self-report. Correlates included in the regression models were selected based on literature review; variables were also included in the model if *p* < 0.10 in the bivariate association, while the final model included variables significant at *p* < 0.05. Categories of analysis included demographic characteristics, sexual-risk behaviours, HIV status, circumcision (MSM and male PWID), drug use behaviours (not exclusive to injection for MSM and FSW), access to comprehensive HIV prevention services, stigma/discrimination and past experiences with physical or sexual violence (rape). Descriptive analysis for aggregate estimates was conducted using RDS-Analyst [19] and logistic regression was conducted using SAS version 9.4 (SAS Institute, Cary, NC, USA).

## Results

Table 1 presents the unweighted pooled demographic characteristics of the survey participant. There were 1432 MSM, 1240 FSW and 492 PWID enrolled in the surveys distributed across the three cities as follows: Maputo (MSM: 34.6%, FSW: 32.2%, PWID: 71.8%), Beira (MSM: 40.7%, FSW: 33.2%), Nampula/Nacala (MSM: 24.7%, FSW: 34.6%, PWID: 28.3%). Male PWID made up the overwhelming majority of the sample population (94.9%). The majority of MSM and FSW were less than 24 years of age, at 79.2 and 71.6%, respectively, while only 18.7% of PWID were young. Most survey participants were single or never married (MSM: 83.9%, FSW: 64.7%, PWID: 58.7%); of note, 7.1% of MSM were married or cohabitating with women, while 3.4% of MSM were married or cohabitating with men. Across all three populations, the majority of participants had secondary education or higher (MSM: 83.3%, FSW: 63.0%, PWID: 57.2%). Among MSM participants, 59.9% were employed while among FSW participants, 23.8% reported additional employment aside from sex work; employment status was not assessed for PWID. Close to two-thirds of MSM participants and male PWID participants were circumcised, 64.3 and 65.7% respectively. Most FSW participants reported ever being pregnant (69.2%).

**Table 1:** Unweighted pooled demographic characteristics of Men who have sex with men (MSM), Female sex workers (FSW) and People who inject drugs (PWID), 2010–2014

Demographic characteristics	MSM (***N*** = 1432)		FSW (***N*** = 1242)		PWID (***N*** = 492)	
	n	%	n	%	n	%
Survey City						
Maputo	496	34.6	400	32.3	353	71.8
Beira	583	40.7	411	33.2	–	–
Nampula/Nacala^a^	353	24.7	429	34.6	139	28.3
Male Sex (PWID only)	–	–	–	–	467	94.9
Age						
Median (min-max)	21 (18–59)		21 (15–53)		32 (18–60)	
15–24 (FSW), 18–24 (MSM, PWID)	1134	79.2	888	71.6	92	18.7
25+	298	20.8	352	28.4	400	81.3
Relationship status						
Single or never married	1198	83.9	798	64.5	289	58.7
Married/cohabitating (FSW, PWID)			87	7.0	103	20.9
Married/cohabitating, with woman (MSM only)	102	7.1	–	–	–	–
Married/cohabitating with man (MSM only)^b^	48	3.4	–	–	–	–
Other (widowed, divorced or separated)	80	5.6	352	28.5	100	20.3
Education Level						
No education or Primary level	238	16.7	458	37.0	210	42.8
Secondary education or higher	1191	83.3	779	63.0	281	57.2
Currently employed (FSW and PWID only)^c^	855	59.9	294	23.8	–	–
Circumcised (MSM and male PWID only)	919	64.3			307	65.7
Ever Pregnant (FSW only)	–	–	856	69.2	–	–

^a^ MSM and PWID surveys were conducted in the neighboring cities of Nampula/Nacala; FSW survey was conducted in Nampula only

^b^ 2 MSM living with men were legally married to a woman

^c^ FSW employment refers to work aside from sex work

RDS-weighted aggregate estimates of self-reported symptoms and STIs among MSM, FSW and PWID survey participants are presented in Table 2. Among MSM participants, 5.8% (95% CI: 3.4–8.2) reported penile discharge, 6.6% (95% CI: 4.0–9.2) reported a sore or ulcer near the anus or penis, and 5.0% (95% CI: 3.5–6.5) reported a previous STI diagnosis; as such 11.9% (95% CI: 7.8–16.0) of MSM participants had a self-reported STI. For FSW participants pooled across the three survey cities, vaginal discharge was reported by 26.7% (95% CI: 19.0–34.5), while 9.1% (95% CI: 1.6–16.7) reported a sore or ulcer near the vagina and 11.2% (95% CI: 7.1–15.2) reported having been previously diagnosed with an STI; there were 33.6% of FSW participants with self-reported STIs (95% CI: 26.0–41.3). Among PWID participants – the majority of whom were men (94.9%) – 13.0% (95% CI: 9.6–16.3) reported an abnormal discharge from the vagina, anus or penis, 9.7% (95% CI: 6.7–12.6) reported a sore or ulcer near the vagina, penis or anus, and 14.2% (95% CI: 11.0–17.4) reported being diagnosed with an STI; self-reported STI was reported by 22.0% (95% CI: 17.0–27.0) of participants.

**Table 2:** Aggregate RDS-weighted self-reported Sexually Transmitted Infections among Men who have sex with men (MSM), Female Sex Workers (FSW) and people who inject drugs (PWID) by survey city in Mozambique, 2011–2014

		Maputo			Beira			Nampula/Nacala*			Total		
Population	Symptom	n: Crude	%: Crude	RDS-weighted (95% CI)	n: Crude	%: Crude	RDS-weighted (95% CI)	n: Crude	%: Crude	RDS-weighted (95% CI)	n: Crude	%: Crude	RDS-weighted Aggregate (95% CI)
MSM		***N*** **= 496**			***N*** **= 583**			***N*** **= 353**			**N = 1432**		
	Abnormal discharge	24	4.8	5.4 (2.1–8.8)	28	4.8	5.2 (1.9–8.4)	22	6.2	7.6 (3.3–12.0)	74	5.2	5.8 (3.4–8.2)
	Sore or ulcer	32	6.5	5.6 (2.0–9.2)	71	12.2	11.4 (6.7–16.0)	20	5.7	5.8 (1.1–10.5)	123	8.6	6.6 (4.0–9.2)
	Diagnosis	20	4.0	4.0 (2.3–5.8)	28	4.8	3.6 (0.0–7.2)	31	8.8	9.5 (5.5–13.4)	79	5.5	5.0 (3.5–6.5)
	**STI self-report**	**54**	**10.9**	**10.6 (4.6–16.7)**	**89**	**15.3**	**14.3 (9.9–18.7)**	**45**	**12.8**	**14.0 (9.2–18.8)**	**188**	**13.1**	**11.9 (7.8–16.0)**
FSW		***N*** **= 400**			***N*** **= 411**			***N*** **= 429**			***N*** **= 1240**		
	Abnormal discharge	93	23.3	24.8 (10.0–39.5)	151	36.9	36.6 (29.4–43.8)	85	19.8	20.9 (14.1–27.8)	329	26.5	26.7 (19.0–34.5)
	Sore or ulcer	27	6.8	7.2 (−0.8–22.1)	51	12.4	13.0 (8.7–17.3)	41	9.6	9.2 (4.4–14.0)	119	9.6	9.1 (1.6–16.7)
	Diagnosis	30	7.5	9.4 (2.4–16.3)	35	8.5	10.3 (5.1–15.6)	69	16.1	15.7 (9.7–21.6)	134	10.8	11.2 (7.1–15.2)
	**STI self-report**	**106**	**26.5**	**29.9 (15.4–44.5.0)**	**171**	**41.6**	**42.9 (35.5–50.3)**	**119**	**27.7**	**31.7 (25.2–38.5)**	**396**	**31.9**	**33.6 (26.0–41.3)**
PWID		**N = 353**						***N*** **= 139**			**N = 492**		
	Abnormal discharge	30	8.5	8.6 (3.9–13.4)	–	–	–	35	25.2	27.4 (16.1–38.7)	65	13.2	13.0 (9.6–16.3)
	Sore or ulcer	21	6.0	5.7 (4.2–7.1)	–	–	–	29	20.9	22.7 (13.7–31.6)	50	10.2	9.7 (6.7–12.6)
	STI Diagnosis	30	8.5	7.2 (2.4–12.0)	–	–	–	47	33.8	36.7 (24.6–48.9)	77	15.7	14.2 (11.0–17.4)
	**STI self-report**	**46**	**13.0**	**11.7 (5.9–17.4)**	**–**	**–**	**–**	**71**	**51.1**	**55.5 (43.4–67.7)**	**117**	**23.8**	**22.0 (17.0–27.0)**

Note: Symptoms for men who have sex with men (MSM) and people who inject drugs (PWID) refer to last 12 months, while female sex workers (FSW) refers to last 6 months

^a^MSM and PWID surveys were conducted in the neighboring cities of Nampula/Nacala; FSW survey was conducted in Nampula only

### Correlates of STI self-report among MSM

When controlling for potential confounders in the multiple regression analysis, MSM participants who had been circumcised (aOR = 1.9, 95% CI: 1.3–2.6; *p* < 0.001), HIV positive (aOR = 2.0, 95% CI: 1.2–3.4; *p* = 0.009), reported illicit drug use (aOR = 2.2, 95% CI: 1.4–3.6; *p* = 0.001), engaged in receptive anal sex (aOR = 1.4, 95% CI: 1.0–2.0; *p* = 0.043), and reported non-condom use with their last male partner (aOR = 1.8, 95% CI: 1.2–2.5; *p* = 0.001) had greater odds of STI self-report, as presented in Table 3.

**Table 3:** Unweighted pooled estimate of risk factors associated with self-reported Sexually Transmitted Infections among Men who have sex with men (*n* = 1432), Mozambique 2012

Variables	Prevalence		Crude Odds Ratio			Adjusted Odds Ratio		
	n/N	%	OR	95% CI	***p***-value	aOR	95% CI	***p***-value
Survey City								
Maputo	54/496	10.9	0.8	0.5–1.3	0.104			
Beira	89/583	15.3	1.2	0.8–1.8	0.058			
Nampula/Nacala	45/353	12.8	REF					
Age (years)								
18–24	127/1134	11.2	REF			REF		
25 and over	61/298	20.5	2.0	1.5–2.9	**< 0.001**	1.2	0.8–1.8	0.395
Relationship status								
Single or never married	134/1198	11.2	REF			REF		
Married/co-habituating, with woman	29/102	28.4	3.2	2.0–5.0	**0.008**	1.8	1.0–3.2	0.273
Married/cohabitating with man^a^	9/48	18.8	1.8	0.9–3.9	0.988	1.6	0.7–3.7	0.672
Other (widowed, divorced or separated)	16/80	20.0	2.0	1.1–3.5	0.749	1.4	0.7–2.6	0.893
Education Level								
No education or Primary education	41/238	17.2	1.5	1.0–2.2	**0.043**	1.2	0.8–1.8	0.463
Secondary education or higher	147/1191	12.3	REF			REF		
Employment Status								
Employed	134/855	15.7	1.8	1.3–2.5	**< 0.001**	1.3	0.9–1.8	0.242
Unemployed	54/573	9.4	REF			REF		
Circumcised								
Yes	99/919	10.8	REF			REF		
No	89/510	17.5	1.8	1.3–2.4	**< 0.001**	1.9	1.3–2.6	**< 0.001**
HIV infection								
Positive	34/114	29.8	3.1	2.0–4.9	**< 0.001**	2.0	1.2–3.4	**0.009**
Negative	151/1262	12.0	REF			REF		
Comprehensive prevention services**								
Yes	71/467	15.2	1.3	0.9–1.8	0.111			
No	117/962	12.2	REF					
Binge drinking (6 or more alcoholic drinks per event)								
Yes	77/473	16.3	1.4	1.1–2.0	**0.022**	1.3	0.9–1.8	0.199
No	111/912	11.8	REF			REF		
Illicit drug use								
Yes	30/139	21.6	2.0	1.3–3.1	**0.002**	2.2	1.4–3.6	**0.001**
No	158/1290	12.3	REF			REF		
Physical violence								
Yes	10/51	19.6	1.6	0.8–3.3	0.170			
No	178/1377	12.9	REF					
Sexual violence								
Yes	6/18	33.3	3.4	1.3–9.1	**0.016**	1.3	0.4–4.3	0.720
No	181/1409	12.9	REF			REF		
Experienced stigma								
Yes	23/129	17.8	1.5	0.9–2.4	0.101			
No	165/1300	12.7	REF					
Concurrent male and female partner								
Yes	109/736	14.8	1.4	1.0–1.8	**0.057**	1.4	1.0–1.9	0.088
No	79/693	11.4	REF			REF		
Receptive anal sex, with man								
Yes	79/523	15.1	1.3	1.0–1.8	**0.098**	1.4	1.0–2.0	**0.043**
No	109/906	12.0	REF			REF		
Insertive anal sex, with man								
Yes	163/1219	13.4	1.1	0.7–1.8	0.562			
No	25/210	11.9	REF					
Condom use at last sexual encounter, with man								
No Condom	114/1021	11.2	1.8	1.3–2.4	**< 0.001**	1.8	1.2–2.5	**0.001**
Yes Condom	74/400	18.0	REF			REF		
Paid or received sex in exchange for money, with man								
Yes	88/636	13.8	1.1	0.8–1.5	0.507			
No	100/791	12.6	REF					

Notes

^a^ 2 men who have sex with men (MSM) living with men were legally married to a woman

**Comprehensive prevention services refers to peer education & Information education and communication (IEC) materials

### Correlates of STI self-report among FSW

Table 4 presents the unweighted pooled estimates of risk factors associated with self-reported STIs among FSW. In the multivariable analysis, when controlling for potential confounders, FSW participants living in Beira (aOR = 2.0, 95% CI: 1.5–2.8, *p* < 0.001), currently married (aOR = 2.0, 95% CI: 1.2–3.3, *p* = 0.005), having employment aside from sex work (aOR = 1.5, 95% CI: 1.1–2.0, *p* = 0.007), having experienced physical violence (aOR = 1.5, 95% CI: 1.1–2.2, *p* = 0.023), having experienced sexual violence (aOR = 2.0, 95% CI: 1.3–2.9, *p* = 0.001), reporting drug use (aOR = 4.0, 95% CI: 1.5–10.5, *p* = 0.005), having access to comprehensive HIV prevention services (aOR = 1.4, 95% CI: 1.0–2.0, *p* = 0.031), reporting non-condom use with last client (aOR = 1.4, 95% CI: 1.0–1.9; *p* = 0.030), and having had concurrent sexual relationship with a non-client steady partner while engaging in sex work (aOR = 1.4, 95% CI: 1.1–1.9, *p* = 0.007) had greater odds of STI self-report.

**Table 4:** Unweighted pooled estimates of risk factors associated with self-reported Sexually Transmitted Infections among Female Sex Workers (N = 1242), Mozambique 2012

Variables	Prevalence		Crude Odds Ratio			Adjusted Odds Ratio		
	n/N	(%)	OR	95% CI	p-value	aOR	95% CI	p-value
Survey City								
Maputo	106/400	26.5	0.9	0.7–1.3	0.006	1.1	0.8–1.6	0.126
Beira	171/411	41.6	1.9	1.4–2.5	**< 0.001**	2.0	1.5–2.8	**< 0.001**
Nampula	119/429	27.7	REF			REF		
Age (years)								
15–24	279/888	31.4						
25 and over	117/352	33.2						
Marital Status								
Single or never married	239/798	30.0	REF			REF		
Married/Co-habiting	42/87	48.3	2.2	1.4–3.4	**0.001**	2.0	1.2–3.3	**0.005**
Other (widowed, divorced or separated)	115/352	32.7	1.1	0.9–1.5	0.101	1.0	0.8–1.4	0.079
Education Level								
No education or Primary level education	154/458	33.6				1.0	0.8–1.4	0.750
Secondary education or higher	242/779	31.1						
Work aside from sex work								
Other work	122/294	41.5	1.7	1.3–2.3	**< 0.001**	1.5	1.1–2.0	**0.007**
No other work	274/943	29.1	REF			REF		
Binge drinking (6 or more alcoholic drinks per event)								
Yes	125/334	37.4	1.4	1.1–1.8	**0.013**	1.2	0.9–1.6	0.153
No	270/901	30.0	REF			REF		
Physical Violence^a^								
Yes	78/172	45.4	2.0	1.4–2.7	**< 0.001**	1.5	1.1–2.2	**0.023**
Never	316/1061	29.8	REF			REF		
Sexual Violence^a^								
Yes	71/138	51.5	2.5	1.8–3.6	**< 0.001**	2.0	1.3–2.9	**0.001**
No	325/1098	29.6	REF			REF		
Illicit drug use								
Yes	16/24	66.7	4.4	1.9–10.3	**< 0.001**	4.0	1.5–10.5	**0.005**
No	380/1213	31.3	REF			REF		
HIV infection								
Positive	123/341	36.1	1.3	1.0–1.7	**0.060**	1.2	0.9–1.6	0.253
Negative	273/896	30.5	REF			REF		
Comprehensive prevention services**								
Yes	80/207	38.7	1.4	1.0–1.9	**0.025**	1.4	1.0–2.0	**0.031**
No	316/1030	30.7	REF			REF		
Condom use with last client								
No condom	123/316	38.9	1.5	1.2–2.0	**0.002**	1.4	1.0–1.9	**0.030**
Condom	271/919	29.5	REF			REF		
Concurrent stable romantic partner & sex work								
Yes	184/485	37.9	1.6	1.2–2.0	**< 0.001**	1.4	1.1–1.9	**0.007**
No	210/745	28.2	REF			REF		

**Comprehensive prevention services refers to peer education & Information education and communication (IEC) materials

### Correlates of STI self-report among PWID

There were greater odds of self-reported STI among PWID who were living in Nampula/Nacala (aOR = 4.8, 95% CI: 2.3–10.3; *p* < 0.001), had access to HIV prevention services (aOR = 2.3, 95% CI: 1.2–4.5; *p* < 0.011) and reported receiving drugs in exchange for money (aOR = 2.2, 95% CI: 1.0–4.7; *p* = 0.40), as presented in Table 5.

**Table 5:** Unweighted pooled estimate of risk factors associated with self-reported syndromic STIs among People Who Inject Drugs (*N* = 492), Mozambique 2014

Variables	Prevalence		Crude Odds Ratio			Adjusted Odds Ratio		
	n/N	(%)	OR	95% CI	***p***-value	aOR	95% CI	***p***-value
Survey City								
Maputo	46/353	13.0	7.0	4.4–11.0	**< 0.001**	REF		
Nampula/Nacala	71/139	51.1				4.8	2.3–10.3	**< 0.001**
Sex								
Female	6/25	24.0	1.0	0.4–2.6	0.979	1.4	0.4–5.0	0.576
Male	111/467	23.8	REF			REF		
Age (years)								
18–24	32/92	34.8	2.0	1.2–3.2	**0.007**	1.0	0.5–2.3	0.955
25 and over	85/400	21.3	REF			REF		
Marital Status								
Single or never married	54/289	18.7	REF					
Married/Co-habiting	34/103	33.0	2.1	1.3–3.6	0.056	1.6	0.8–3.4	0.947
Other (widowed, divorced or separated)	29/100	29.0	1.8	1.1–3.0	0.448	2.4	1.2–5.0	0.055
Education Level								
No education or Primary level education	37/210	17.6	REF					
Secondary education or higher	80/281	28.5	1.9	1.2–2.9	**0.006**	0.9	0.5–1.7	0.849
Circumcised (Males only, *n* = 193)								
Yes	89/307	29.0	2.6	1.5–4.3	**< 0.001**			
No	22/160	13.8	REF					
HIV infection								
Positive	44/204	21.6	0.8	0.5–1.2	0.222	1.6	0.8–3.0	0.197
Negative	64/241	26.6	REF			REF		
Comprehensive prevention services**								
Yes	41/78	52.6	4.9	3.0–8.2	**< 0.001**	2.3	1.2–4.5	**0.011**
No	76/414	18.4	REF			REF		
Phyisical Violence^a^								
Yes	22/77	28.6	1.3	0.8–2.3	0.283			
Never	95/415	22.9	REF					
Sexual Violence^a,b^								
Yes	1/6	16.7	0.6	0.1–5.5	0.684			
No	116/486	23.9	REF					
Experienced stigma^a^								
Yes	39/84	27.4	3.8	2.3–6.2	**< 0.001**	1.0	0.5–1.9	0.974
No	72/387	14.7	REF			REF		
Condom use at last sexual encounter								
Yes	36/173	20.8	1.7	0.9–3.3	0.957	1.0	0.4–2.4	0.888
No	67/214	31.3	2.9	1.6–5.5	**< 0.001**	1.1	0.5–2.7	0.726
Not sexually active	14/104	13.5	REF			REF		
Received drugs in exchange for sex								
Yes	27/67	40.3	2.5	1.5–4.3	**0.001**	2.2	1.0–4.7	**0.040**
No	90/424	21.2	REF			REF		

^a^ Last 12 m (PWID)

^b^ Fishers exact test

**Comprehensive prevention services refers to peer education & Information education and communication (IEC) materials

## Discussion

STI self-report was higher among the KP groups compared to the general population (MSM: 11.9%, FSW: 33.5%, PWID: 22.0% vs Men: 5.0% and Women: 7.0%). The high-burden of STIs among KPs are consistent with other studies and underscores the importance of integrating STI prevention efforts in KP prevention and treatment services [21, 22]. This is especially important among FSW where a third of the FSW participants self-reported STI, thus highlighting the generalized risk of STI among this entire population group.

The results from MSM show that STI self- report was associated with receptive (vs insertive) sex, which is consistent with findings from other studies in the region [23]. Other risk factors consistent with the literature included circumcision, which may be a result of decreased perception of risk. Physical and sexual violence were also major risk factors for STI among FSW and the socio-cultural dynamics contributing to this vulnerability, such as gender power inequalities, economic disparities, and criminalization of sex work, have been explored previously [21, 24, 25].

The results suggest that having other work aside from sex work is associated with more risk, however this requires further investigation given that it is contrary to findings among FSW in Uganda where having employment outside of sex work was considered a protective factor [21].

While HIV infection was only significantly associated with STI self-report among MSM [11, 12], the similar modes of sexual transmission necessitate a need for concentrated efforts to encourage safer sexual behaviours, such as condom use. This is especially important when considering the greater odds of STI-self report among FSW reporting non-condom use with their last client and MSM reporting non-condom use with their last male partner. Given the dynamics of bridging populations - a subgroup of people who have sexual contact with both KP and the general population such as MSM married to women, clients of FSW and non-client sexual partners of FSW - non-condom use represents a potential public health risk to the wider population [2]. Access to HIV prevention services was associated with STI-self report among FSW and PWID, although this may be a result of having symptoms which put one in contact with health services.

Our findings draw attention to the intersectionality of key population groups and their risk behaviours. For example, sex work was associated with STI self-report for PWID, while illicit drug use was a risk factor among both FSW and MSM; these overlapping risk profiles were also found in other studies [22, 24]. Our results emphasize that treatment and prevention efforts are limited when only considering the primary risk behaviour of a population while isolating others. Any efforts targeted to KP must adopt a people-centred approach to address overlapping risk behaviours.

Although the sample size of female PWID in our study was too small to perform meaningful analysis (*n* = 25), other studies have pointed to the unique vulnerabilities of female PWID [26]. Qualitative studies among female PWID in Mozambique would provide more information about the gendered nature of risk factors in this group.

Finally, there were greater odds of self-report among FSW residing in Beira and PWID residing in Nampula/Nacala. Limited resources require the geographic prioritization of efforts based on evidence and require the standardized implementation of quality treatment and prevention services.

Since the implementation of these BBS surveys, the National HIV Program has scaled up prevention, care and treatment efforts for KPs in Mozambique. In 2016, National Guidelines were published that aimed to integrate HIV prevention and treatment services for KPs into the health sector [27]. These included the creation of standardized package of services for KPs with structural, biomedical and behavioural interventions, including STI screening, diagnosis and treatment. The guidelines commit to offering evidence-based quality services with a people-centred approach free from stigma and discrimination. The guidelines also aimed to strengthen the linkage between community and clinical services to ensure HIV testing among these hard to reach populations. The importance of STI prevention and control among KP was further outlined in the 2018–2021 National Strategic Plan for the Prevention and Control of STIs [6]. Future BBS surveys will be able to assess the extent KPs engagement with the health system, experiences of stigma and STI self-report.

Although this is the first analysis of risk factors associated with STIs among MSM, FSW and PWID in Mozambique, there are several limitations to consider. First, the reliance of self-reported STI symptoms, rather than laboratory testing of common and treatable STIs such as syphilis, chlamydia, and gonorrhoea, could have potentially underestimated STI prevalence by excluding asymptomatic cases. In addition, symptoms such as, discharge, may not necessarily have been the result of an STI. Second, not all survey measures were included in the three surveys (e.g. stigma/discrimination), thus it is not possible to compare risk factors across the different population groups. Additionally, the survey is also subject to the limitations to the survey design such as social desirability, interviewer and recruitment bias. Similar to other cross-sectional surveys, it is also not possible to assess temporality. For example, it is not possible to determine if having access to health services brought PWID and FSW in contact with STI diagnosis or if perhaps having an STI symptom may have caused one to seek out services. Finally, the analysis pooled results from across the survey cities thus severing social networks and chains. As a result, these findings need to be interpreted with caution and cannot be generalized to the full MSM, FSW and PWID in the survey cities nor to KP in Mozambique. Despite the limitations, however, the results of the analysis point to the high burden of STIs among key population groups in Mozambique and provide the evidence needed to advocate for comprehensive and integrated policies and health systems approaches to improve STI screening and case management among high-risk groups in Mozambique.

## Conclusion

The high burden of STIs in KP highlights the need for integrated HIV and STI prevention, outreach and treatment services that address the overlapping risk profiles of individuals; this is specifically relevant for harm reduction interventions targeted to PWID that must also include STI screening and promote condom use. Future survey and surveillance studies should consider including laboratory testing of STIs in order to identify and treat asymptomatic cases. Finally, a robust public health response would include the creation of a national STI surveillance system, for better screening and diagnostic procedures. Monitoring the prevalence of STIs, especially among KP, must be seen as an important element of any efforts toward HIV epidemic control.

## Data Availability

The dataset analysed for the current study are fully available from the Data Management Unit of the Mozambique National Institute of Health (INS) data repository for researchers who meet the criteria for access to confidential data following the submission of a concept note. For information, please visit: www.ins.gov.mz or contact: secretaria@ins.gov.mz.

## Abbreviations

BBS: Biological and behavioral Survey
KP: Key populations
FSW: Female sex workers
HIV: Human immunodeficiency virus
MSM: Men who have sex with men (MSM)
PWID: People who inject drugs (PWID)
RDS: Respondent-driven sampling
RDS-A: RDS-Analyst
STI: Sexually transmitted Infection
WHO: World Health Organization
